# Lipoprotein(a) in Cardiovascular Diseases

**DOI:** 10.1155/2013/650989

**Published:** 2012-12-30

**Authors:** Michele Malaguarnera, Marco Vacante, Cristina Russo, Giulia Malaguarnera, Tijana Antic, Lucia Malaguarnera, Rita Bella, Giovanni Pennisi, Fabio Galvano, Alessandro Frigiola

**Affiliations:** ^1^International Ph. D. Program in Neuropharmacology, University of Catania, 95123 Catania, Italy; ^2^Department of Senescence, Urological, and Neurological Sciences, University of Catania, 95126 Catania, Italy; ^3^Department of Biomedical Sciences, University of Catania, 95124 Catania, Italy; ^4^Department of Neurosciences, University of Catania, 95123 Catania, Italy; ^5^Department of Biological Chemistry, Medical Chemistry, and Molecular Biology, University of Catania, 95123 Catania, Italy; ^6^Pediatric Cardiology and Cardiac Surgery Department, Guch Unit, IRCCS Policlinico San Donato, 20097 Milan, Italy

## Abstract

Lipoprotein(a) (Lp(a)) is an LDL-like molecule consisting of an apolipoprotein B-100 (apo(B-100)) particle attached by a disulphide bridge to apo(a). Many observations have pointed out that Lp(a) levels may be a risk factor for cardiovascular diseases. Lp(a) inhibits the activation of transforming growth factor (TGF) and contributes to the growth of arterial atherosclerotic lesions by promoting the proliferation of vascular smooth muscle cells and the migration of smooth muscle cells to endothelial cells. Moreover Lp(a) inhibits plasminogen binding to the surfaces of endothelial cells and decreases the activity of fibrin-dependent tissue-type plasminogen activator. Lp(a) may act as a proinflammatory mediator that augments the lesion formation in atherosclerotic plaques. Elevated serum Lp(a) is an independent predictor of coronary artery disease and myocardial infarction. Furthermore, Lp(a) levels should be a marker of restenosis after percutaneous transluminal coronary angioplasty, saphenous vein bypass graft atherosclerosis, and accelerated coronary atherosclerosis of cardiac transplantation. Finally, the possibility that Lp(a) may be a risk factor for ischemic stroke has been assessed in several studies. Recent findings suggest that Lp(a)-lowering therapy might be beneficial in patients with high Lp(a) levels. A future therapeutic approach could include apheresis in high-risk patients in order to reduce major coronary events.

## 1. Introduction

Cardiovascular diseases cause 3% of all deaths in North America being the most common cause of death in European men under 65 years of age and the second most common cause in women. These facts suggested us to consider new strategies for prediction, prevention, and treatment of cardiovascular disease [1]. Inflammatory mechanisms play a central role in the pathogenesis of atherosclerosis and its complications [2]. It has been demonstrated that atherogenic lipoproteins such as apo(B-100), oxidized low-density lipoprotein (LDL), remnant lipoprotein (beta-VLDL), and lipoprotein(a) play a critical role in the proinflammatory reaction. High-density lipoprotein (HDL) is antiatherogenic lipoproteins that exert anti-inflammatory functions [3–5]. Plasma LDL cholesterol is a well-established predictor of coronary artery disease (CAD), and many observations have pointed out that Lp(a) and apolipoprotein(a) (apo(a)) levels may be risk factors for cardiovascular diseases (CVD) [6–8].

## 2. Native Lp(a)

Lp(a) is an LDL-like molecule consisting of an apolipoprotein B-100 (apo(B-100)) particle attached by a disulphide bridge to apo(a). Lp(a) plasma concentrations are controlled by the apo(a) gene located on chromosome 6q26-27 [9]. The unique character of Lp(a) is based on the apo(a) highly glycosylated protein structurally homologous to plasminogen [10]. Several published data indicated the existence of unbound forms of apo(a) in blood and urinary excretion of apo(a) fragments [11, 12]. Animal experiments showed that apo(a) serves as a distinctive marker of Lp(a) and represents an atherogenic component of Lp(a) [13]. Furthermore, apo(a) has also been reported to be correlated to coronary artery disease as well as renal disease [14–16]. Dissociation of apo(a) may lead to the exposure of an additional lysine-binding site, increasing the affinity of free apo(a) for plasmin modified fibrin, thus impeding fibrinolysis [17]. Apo(a) is a member of a family of “kringle" containing proteins, such as plasminogen, tissue plasminogen activator (tPA), prothrombin, factor XII, and macrophage stimulating factor (MSF). Lp(a) shares a high degree of sequence identity with plasminogen. These similarities could explain the role of Lp(a) in thrombogenesis and as a proinflammatory factor [18]. Native Lp(a) has been shown to enhance the expression of adhesion molecules [13, 19–23]. Because of the structural homology with plasminogen, Lp(a) might have important antithrombolytic properties, which could contribute to the pathogenesis of atherothrombotic disease. For example, Lp(a) binding to immobilised fibrinogen and fibrin results in the inhibition of plasminogen binding to these substrates [24, 25]. In addition, Lp(a) competes with plasminogen for its receptors on endothelial cells, leading to diminished plasmin formation, thereby delaying clot lysis and favouring thrombosis. The high affinity of Lp(a) for fibrin provides a mechanistic basis for their frequent colocalization in atherosclerotic plaques [26, 27]. Moreover Lp(a) induces the monocyte chemoattractant (CC chemokine I-309), which leads to the recruitment of mononuclear phagocytes to the vascular wall [28, 29].

## 3. Oxidized Lp(a) 

Lp(a) particles can suffer oxidative modification and scavenger receptor uptake, with cholesterol accumulation and foam cell formation [30], leading to atherogenesis. Oxidation of LDL and Lp(a) affects the catabolism of the lipoproteins, including changes in receptor recognition, catabolic rate, retention in the vessel wall, and propensity to accelerate atherosclerosis. Oxidative modification of apo(a) may have an influence on Lp(a) recognition by scavenger receptors of macrophages. Some studies showed that Lp(a) particles are prone to oxidation and that the increased risk of coronary artery disease associated with elevated Lp(a) levels may be related in part to their oxidative modification and uptake by macrophages, resulting in the formation of macrophage-derived foam cells [31]. The oxidative form of Lp(a) (ox-Lp(a)) might attenuate fibrinolytic activity through the reduction of plasminogen activation, might enhance PAI-1 production in vascular endothelial cells, and might impair endothelium-dependent vasodilation. Particularly, the role of ox-Lp(a) is linked to macrophages that take up ox-Lp(a) via scavenger receptor as well as oxidized LDL. Lp(a) particles are susceptible to oxidative modification and scavenger receptor uptake, leading to intracellular cholesterol accumulation and foam cell formation, which contributes further to atherogenesis [25, 32]. Morishita et al. demonstrated increased values of ox-Lp(a) in patients with coronary artery disease [33]. A study of autopsy findings demonstrated a deposition of ox-Lp(a) in the vessel margin inside the calcified areas [34]. Probably it was related to the promotion of an antifibrinolytic environment, foam cell formation, generation of a fatty streak, and an increase in smooth muscle cells. Moreover ox-Lp(a) is a potent stimulus of monocyte adhesion to endothelial cells, thus contributing to atherogenic changes in human blood vessels. Komai et al. compared the effects of oxidized lipoproteins and no oxidized lipoprotein on the progression of atherosclerosis. It was investigated the mitogenic actions of Lp(a) and ox-Lp(a) on human vascular smooth muscle cells (VSMC). The results were that Lp(a) significantly stimulated the growth of human VSMC in a dose-dependent manner, whereas ox-Lp(a) showed a stronger stimulatory action on VSMC growth than native Lp(a). This study demonstrated that ox-Lp(a) has a more potent effect than native Lp(a) in developing atherosclerosis diseases [35].

## 4. Glycated Lp(a) 

Nonenzymatic glycation of lipoprotein may contribute to the premature atherogenesis in patients with diabetes mellitus by diverting lipoprotein catabolism from nonatherogenic to atherogenic pathways. It has been observed that the proportion of apo (B-100) in glycated form was significantly higher in diabetic patients than in nondiabetic controls, and equally that plasma Lp(a) levels might be increased in diabetic patients [36]. Anyway, glycation does not appear to significantly enhance the atherogenic potential of unmodified Lp(a) [37]. The kringle of apo(a) is homologous to the kringle IV of plasminogen, and each of these kringles contains a potential site of N-linked glycosylation. The carbohydrate content of apo(a) has been determined and represents approximately 28% by weight of the protein. Peripheral levels of Lp(a) have been examined in a number of studies involving diabetic patients because Lp(a) concentration is associated with a high-risk of coronary heart disease, and diabetic patients are prone to develop coronary heart disease. It has been demonstrated that glycation enhances the production of PAI-1 and attenuates the synthesis of t-PA induced by Lp(a) in arterial and venous endothelial cells (EC). The formation of advanced glycation end-products (AGEs) and EC-mediated oxidative modification may contribute to the alterations of the generation of PAI-1 and t-PA induced by glycated Lp(a) [13]. The combination of hyperglyaemia and hyperlipoprotein(a) may reduce EC-derived fibrinolytic activity, which may promote the development of thrombosis and atherosclerosis in subjects with diabetes [36].

## 5. Atherogenic and Proinflammatory ****Mechanisms of Lp(a)

### 5.1. Lp(a) and Endothelial Dysfunction

As the atherosclerotic plaque progresses, growth factors and cytokines secreted by macrophages and foam cells in the plaque stimulate vascular smooth muscle cell growth and interstitial collagen synthesis [37]. Moreover, the apo(a) component of Lp(a) has been shown to enhance the expression of ICAM-1 [13]. Thus, these effects on endothelial cell function may provide mechanisms by which Lp(a) contributes to the development of atherosclerotic lesions. Reduction in nitric oxide (NO) availability also initiates the activation of matrix metalloproteinases MMP-2 and MMP-9 [38, 39], and further it reduces inhibition of platelet aggregation [40]. Thus, endothelial dysfunction with reduced NO bioavailability, increased oxidant excess, and expression of adhesion molecules contributes not only to initiation but also to progression of atherosclerotic plaque formation and triggering of cardiovascular events. In vitro studies indicated that Lp(a) enhances the synthesis of PAI-1 by endothelial cells. PAI-1 is the main inhibitor of the fibrinolytic system [41]. Another potentially important action of Lp(a) is the reduction of activation of latent transforming growth factor-*β* (TGF-*β*) by displacing plasminogen from the surfaces of macrophages in atherosclerotic plaques. In the absence of activated TGF-*β*, cytokines might induce smooth muscle cell proliferation and the transformation of these cells to a more atherogenic cellular phenotype [42, 43]. Furthermore, studies on cultured human umbilical vein or coronary artery endothelial cells revealed a novel effect of Lp(a) that was mediated by its apo(a) component: impairment of the barrier function of endothelial cells through cell contraction occurring as a consequence of a rearrangement of the actin cytoskeleton [44]. In 1992, Cohn et al. studied the abnormalities of vascular compliance in hypertension and demonstrated that vasodilation is inhibited by ox-Lp(a). Also, they showed that elevation of ox-Lp(a) may explain the endothelial dysfunction observed in hypertensive patients because ox-Lp(a) enhanced Lp(a)-induced PAI-1 production in vascular endothelial cells [45]. However, the exact role of ox-Lp(a) is still largely unknown, and at the moment a stronger involvement of the ox-Lp(a) in atherosclerotic development and worse evolution in stroke and heart failure are just supposed. The matter of fact is that ox-Lp(a) might cause more pronounced stimulation of superoxide production, whereas native Lp(a) itself caused a moderate, dose-dependent stimulation of superoxide production. Accumulation of native Lp(a) may enhance the stimulation of ox-Lp(a), a more potent atherogenic lipoprotein, in the vessel wall [46].

### 5.2. Inflammation, Atherosclerosis, and Lp(a)

**Figure 1:**
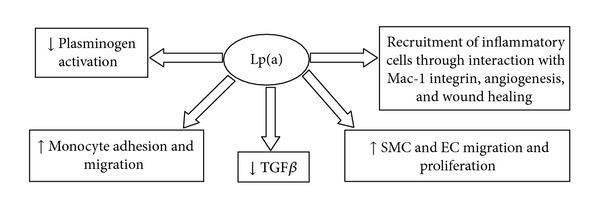
Mechanisms underlying the Lp(a)-induced cardiovascular disease. Lp(a) inhibits the activation of TGF and promotes the proliferation and migration of smooth muscle cells to endothelial cells. Moreover Lp(a) inhibits plasminogen activation and decreases the activity of fibrin-dependent tissue-type plasminogen activator.

Lp(a) may act as a proinflammatory mediator that augments the lesion formation in atherosclerotic plaques [47]. Lp(a) may lead to an inflammatory process by inducing the expression of adhesion molecules on endothelial cells, the chemotaxis of monocytes, and the proliferation of smooth muscle cells [48]. Moreover Lp(a) can augment the production of cytokines by vascular cells, and through the autocrine and paracrine mechanisms, the inflammatory reaction may lead to a vicious cycle resulting in lesion progression [49]. Lp(a) acts on the fibrinolytic system in several ways which include the inhibition of plasminogen binding and activation, thereby impairing fibrinolytic activity and the dissolution of thrombi. High concentrations of Lp(a) might increase the risk of thrombus formation by impeding fibrinolytic mechanisms in the region of the plaque. Some mechanisms are involved into the development of atherosclerosis: Lp(a) inhibits the activation of transforming growth factor (TGF) and contributes to the growth of arterial atherosclerotic lesions by promoting the proliferation of vascular smooth muscle cells and the migration of smooth muscle cells to endothelial cells [50, 51]. Moreover Lp(a) inhibits plasminogen binding to the surfaces of endothelial cells and decreases the activity of fibrin-dependent tissue-type plasminogen activator. Furthermore Lp(a) increases plasminogen activator inhibitor activity in endothelial cells and promotes atherothrombosis [52]. Other functions have been related to recruitment of inflammatory cells through interaction with Mac-1 integrin, angiogenesis, and wound healing (Figure 1). However, individuals without Lp(a) or with very low Lp(a) levels seem to be healthy. Thus plasma Lp(a) is certainly not vital, at least under normal environmental conditions [53–55]. 

## 6. Lp(a) and Cardiovascular Diseases

The relationship between Lp(a) levels and the severity of coronary atherosclerosis in patients with unstable angina or acute myocardial infarction (MI) has been analyzed in several studies with controversial results [77–79]. The potential value of small apo(a) isoforms in predicting severe angiographically demonstrable atherosclerosis remains unclear. Elevated serum Lp(a) is an independent predictor of coronary artery disease (CAD) and myocardial infarction [64, 68, 80]. Motta et al. studied the transient increased serum levels of this lipoprotein during acute myocardial infarction (AMI). The positive correlation between mean Lp(a) values on day 1 and 7 and the size of the necrotic area suggested an atherogenic and prothrombotic role of Lp(a). Moreover, elevated Lp(a) values were related to greater tissue damage. This study suggested that periodical determination of Lp(a) values in subjects with coronary disease may be useful in order to predict further acute vascular events [81]. Lp(a) levels should be a marker of restenosis after percutaneous transluminal coronary angioplasty [82], saphenous vein bypass graft atherosclerosis [83], and accelerated coronary atherosclerosis of cardiac transplantation [84]. Some studies have shown that Lp(a) is not associated with atherosclerosis, and others have demonstrated that a high serum Lp(a) level is a major risk factor for atherosclerosis and progression of coronary artery disease [85–87]. A high serum Lp(a) level may be a high-risk factor for CCSP (clinical coronary stenosis progression) and restenosis after PCI (percutaneous coronary intervention). An elevated Lp(a) concentration is a significant predictor of long-term adverse outcome in AMI patients treated by primary percutaneous transluminal coronary angioplasty [88]. Serum Lp(a) levels ≥ 25 mg/dL are noted in 67% of patients with rapid progression of coronary artery disease but in only 33% of patients without progression of coronary artery disease [89]. Tamura et al. studied the association between serum Lp(a) level and angiographically assessed coronary artery disease progression without new myocardial infarction, reporting a significant association [90]. In patients with serum Lp(a) levels ≥ 30 mg/dL, coronary stenosis progression occurred, and revascularizations for target restenotic lesions or new lesions were performed approximately 7 months after the first myocardial infarction; CCSP occurred in a relatively short period after the first AMI in the high-Lp(a) patients [91]. A meta-analysis demonstrated that Lp(a) levels can be considered as a risk factor for cardiovascular disease [92, 93]. The first study of the association between Lp(a) and a range of cardiovascular endpoints including cognitive and disability indices in the elderly was conducted by Gaw et al. The main finding was that Lp(a) level, although influenced by a number of baseline characteristics, is not a significant predictor of cognitive function or levels of disability but is a predictor of combined cardiovascular events over an average 3.2-year followup [94]. Sandholzer et al. reported that in patients with premature coronary heart disease (CHD), alleles at the apo(a) locus determine risk for CHD through their effect on plasma Lp(a) level. This study suggested that Lp(a) can be considered a primary genetic risk factor for CHD [95]. Several epidemiologic studies have assessed the association between Lp(a) and atherosclerotic disease (Table 1). Many population-based prospective studies had reported a controversial association between Lp(a) levels and CHD risk. Few studies, however, have adequately examined important aspects of the association, such as the size of relative risks in clinically relevant subgroups (such as in men and women or at different levels of established risk factors) [56–63, 65–67, 69, 70, 73, 74, 96–98]. A task force for emerging risk factor assessed the relationship between Lp(a) concentration and risk of major vascular and nonvascular outcomes. In this long-term prospective study, Lp(a) plasma levels and subsequent major vascular morbidity and/or cause-specific mortality were recorded. Lp(a) was weakly correlated with several conventional vascular risk factors, and it was highly consistent within individuals over several years. The risk ratio for CHD, adjusted for age and sex only, was 1.16 per 3.5-fold higher usual Lp(a) concentration (i.e., per 1 SD), and it was 1.13 following further adjustment for lipids and other conventional risk factors. The corresponding adjusted risk ratios were 1.10 for ischemic stroke, 1.01 for the aggregate of nonvascular mortality, 1.00 for cancer deaths, and 1.00 for nonvascular deaths other than cancer. The results showed that there are continuous, independent, and modest associations of Lp(a) concentration with risk of CHD and stroke that appear exclusive to vascular outcomes [99]. The Copenhagen City Heart Study (CCHS) found that extreme Lp(a) levels > 95th percentile predict a 3- to 4-fold increase in risk of myocardial infarction (MI) and absolute 10-year risks of 20% and 35% in high-risk women and men [100]. In this study it was observed larger risk estimate for Lp(a) than most previous studies, most likely because the authors focused on extreme levels, measured levels shortly after sampling, corrected for regression dilution bias, and considered MI rather than ischemic heart disease (IHD). For the first time CCHS provided absolute 10-year risk estimates in the general population for MI and IHD as a function of Lp(a) levels stratified for other risk factors, allowing clinicians to use extreme Lp(a) levels in risk assessment of individual patients [101]. In conclusion, most but not all prospective studies on Lp(a) and risk of CHD have found positive associations, and levels of Lp(a) have also been related to severity of disease. Lp(a) levels differ between ethnic groups, and thus results from one study may not be applicable to other ethnic groups. Recent recommendations stated that Lp(a) screening is not warranted for primary prevention and assessment of cardiovascular risk at present, but that Lp(a) measurements can be useful in patients with a strong family history of cardiovascular disease or if risk of cardiovascular disease is considered intermediate on the basis of conventional risk factors [102]. The European Atherosclerosis Society Consensus Panel [103] have suggested screening for elevated Lp(a) in those at intermediate or high CVD/CHD risk, a desirable level < 50 mg/dL as a function of global cardiovascular risk, and use of niacin for Lp(a) and CVD risk reduction.

## 7. Conclusions

The clinical interest in Lp(a) is largely derived from its role as a cardiovascular risk factor. Although not considered an established risk factor, Lp(a) levels have been associated with cardiovascular disease in numerous studies [72, 104, 105]. Recently Lp(a) serum levels were found to be associated with the severity of aortic atherosclerosis, especially in abdominal aorta, as well as coronary atherosclerosis [106]. Moreover a study by Momiyama et al. [107] demonstrated that elevated Lp(a) has incremental prognostic value in symptomatic patients with coronary artery revascularization [108]. Lp(a) is involved in the development of atherothrombosis and activation of acute inflammation exerting a proatherogenic and hypofibrinolytic effect. Lp(a) plays a critical role in the proinflammatory reaction and can be considered as a common joint among different metabolic systems. Other actions of Lp(a) can be resumed as follows: inhibition of the activation of plasminogen; inhibition of the activation of TGF-*β*; activation of acute inflammation; induction of the expression of adhesion molecules; elevation of the production of cytokines. Moreover Lp(a) is implicated in the activation of endothelial uptake, oxidative modification, and foam cell formation, suggesting that these processes could play an important role in atherosclerosis. Recent findings suggest that Lp(a)-lowering therapy might be beneficial, at least in some subgroups of patients with high Lp(a) levels. A possible future therapeutic approach could include apheresis in high-risk patients with already maximally reduced LDL cholesterol levels in order to reduce major coronary events [72]. However, further studies are needed to define such subgroups with regard to Lp(a) levels, apo(a) size, and the presence of other risk factors. 

## Figures and Tables

**Table 1:** Lp(a) values in patients that developed atherosclerotic disease.

	Cases		Controls			
	*n*	Lp(a) (mg/dL)	*n*	Lp(a) (mg/dL)	Years of follow up	Year of the study
Alfthan et al. [56]						
Males	97	73	148	108		
Females	97	113	121	91	8	1994
Assmann et al. [57]	33	90	828	50	8	1996
Coleman et al. [58]	49	402	192	288	1–9	1992
Cremer et al. [59]	107	180	5124	90	5	1994
Jauhiainen et al. [60]	138	131	130	111	6-7	1991
Klausen et al. [61]	74	124	190	94	8 or 15	1997
Ridker et al. [62]	296	103	296	102.5	5.02	1995
Rosengren et al. [63]	26	277.7	109	172.7	6	1990
Schaefer et al. [64]	233	237	390	195	7–10	1994
Sigurdsson et al. [65]	104	230	1228	170	8.6	1992
Wald et al. [66]	229	85.8	1145	55.6	5–12	1994
Wild et al. [67]						
Males	90	125.1	90	63.5		
Females	44	97.3	44	72.7	13	1997
Bostom et al. [68]	305	>30	3103		12	1994
Bostom et al. [69]	129		2191		15.4	1996
Cantin et al. [70]	116	>30	2156		5	1998
Cressman et al. [71]		38.4	129	16.9	4	1992
Stubbs et al. [71]		>30	266 197		3	1998
Kronenberg and Utermann [72]		32.8	826	8.8	5	1999
Bennet et al. [72]	2047	43.8	3921	40.4	12	2008
Dahlén et al. [73]	62	250.2	124	134.7	11	1998
Cantin et al. [70]	116 with IHD	41	2040	32.7	5	1998
Nguyen et al [74]	9936	32.8	826	8.8	14	1997
Ariyo et al. [72]	3972	4.2			7.4	2003
Hoogeveen et al. [75]	57	12.65	46	9.15		2001
Dirisamer et al. [76]	103	20	103	15		2002
